# Advances in Personalized Targeted Treatment of Metastatic Melanoma and Non-Invasive Tumor Monitoring

**DOI:** 10.3389/fonc.2013.00054

**Published:** 2013-03-19

**Authors:** Dragana Klinac, Elin S. Gray, Michael Millward, Mel Ziman

**Affiliations:** ^1^School of Medical Sciences, Edith Cowan UniversityPerth, WA, Australia; ^2^School of Medicine and Pharmacology, University of Western AustraliaCrawley, WA, Australia; ^3^School of Pathology and Laboratory Medicine, University of Western AustraliaCrawley, WA, Australia

**Keywords:** metastatic melanoma, personalized treatment, targeted therapy, drug resistance, circulating tumor cells

## Abstract

Despite extensive scientific progress in the melanoma field, treatment of advanced stage melanoma with chemotherapeutics and biotherapeutics has rarely provided response rates higher than 20%. In the past decade, targeted inhibitors have been developed for metastatic melanoma, leading to the advent of more personalized therapies of genetically characterized tumors. Here we review current melanoma treatments and emerging targeted molecular therapies. In particular we discuss the mutant BRAF inhibitors Vemurafenib and Dabrafenib, which markedly inhibit tumor growth and advance patients’ overall survival. However this response is almost inevitably followed by complete tumor relapse due to drug resistance hampering the encouraging initial responses. Several mechanisms of resistance within and outside the MAPK pathway have now been uncovered and have paved the way for clinical trials of combination therapies to try and overcome tumor relapse. It is apparent that personalized treatment management will be required in this new era of targeted treatment. Circulating tumor cells (CTCs) provide an easily accessible means of monitoring patient relapse and several new approaches are available for the molecular characterization of CTCs. Thus CTCs provide a monitoring tool to evaluate treatment efficacy and early detection of drug resistance in real time. We detail here how advances in the molecular analysis of CTCs may provide insight into new avenues of approaching therapeutic options that would benefit personalized melanoma management.

## Introduction

Melanoma is an aggressive cutaneous cancer that arises from melanocyte cells within the basal layer of the epidermis. This aggressive malignancy accounts for more than 80% of skin cancer deaths and its incidence is increasing worldwide (Linos et al., 2009). Cutaneous melanoma arises from the transformation of melanocytes by the accumulation of mutations in genes that regulate cell differentiation and proliferation. The disease manifests itself as clinically and genetically distinct subgroups indicating the need for patient-specific treatment strategies.

In the past decade, since the discovery of key mutations and activated pathways that drive the development of melanoma (Davies et al., 2002), new targeted therapies have been developed, with mixed success. In the fore front of these is a molecule that specifically inhibits the mutated BRAF^V600E^ kinase, Vemurafenib, which was approved by the FDA in 2011 as a therapeutic option for treatment of unresectable metastatic melanoma (Chapman et al., 2011). Given the success of this treatment and other treatment advances detailed below, new guidelines for the treatment of melanoma are evolving (Fox et al., 2013). Moreover, deep sequencing analyses have revealed new potential targets and much has been learned about the molecular basis of melanoma genesis. A clearer landscape of the mutation profile of melanoma is emerging and with it new potential therapeutic targets.

## Mutations in Melanoma

The most commonly observed recurrent mutations in melanoma reside within the MAPK pathway. The MAPK/Extracellular signal-regulated kinase (ERK) signaling pathway is commonly activated in melanoma by mutations in BRAF (in 50% of melanomas), NRAS (10–20%), and less frequently in MEK1 and MEK2 (∼8%) (Davies et al., 2002; Curtin et al., 2005; Murugan et al., 2009; Dutton-Regester and Hayward, 2012). Around 70–95% of all BRAF mutations are a V600E substitution, with an alternative V600K in 5–30% of the cases. BRAF and NRAS mutations are usually exclusive with a Q61R substitution in ∼60% of NRAS mutated cases (Colombino et al., 2012).

Mutations in upstream tyrosine kinase receptors such as KIT (10%, mainly in acral and mucosal melanoma), ERBB4 (∼19%) (Prickett et al., 2009), and FGFR2 (∼10%) (Gartside et al., 2009), can activate both the MAPK/ERK and the PI3K/AKT pathways.

Activating mutations in the kinases PI3K (∼3%) and AKT (∼1%) have also been reported, albeit at lower frequencies (Davies et al., 2002; Omholt et al., 2006). More common are mutations or deletions in the tumor suppressor gene PTEN (∼10–27%), responsible for the negative regulation of the PI3K/AKT pathway (Paraiso et al., 2011). Mutations in PREX2 (14%), a negative regulator of PTEN, have been described recently (Berger et al., 2012).

Another tumor suppressor gene commonly altered in melanoma is CDKN2A (∼50%) which regulates the pRB and p53 pathways (Flores et al., 1996). Additional driver mutations in TP53 (∼20%), CDK4 (∼3%), and RB1 (∼3%) have also been described, as well as a hot-spot in the adapter protein TRRAP (4%) (Wei et al., 2011). Furthermore, many mutations have been reported in other components that control cellular proliferation, angiogenesis and apoptosis, including glutamate receptors GRIN2A (33%) (Wei et al., 2011) and GRM3 (16%) (Prickett et al., 2011), G-protein GQNA (50% malignant blue nevi and 46% of uveal melanomas) (Van Raamsdonk et al., 2010), and the kinases MAP3K5 (9%) and MAP3K9 (15%) (Stark et al., 2012). Other genomic aberrations include amplifications in MITF (4%), CDK4 (3%), CCND1 (11%) and TERT (13%), and deletions in CDKN2A (38%) (Hodis et al., 2012).

A recent study described five new genes containing potential driver mutations, PPP6C, RAC1, SNX31, TACC1, STK19, and ARID2. The serine/threonine phosphatase PPP6C which negatively regulates the CCND1 oncogene, appears mutated in 12% of sun-exposed melanomas (Krauthammer et al., 2012), with the R264C substitution in 3% of cases (Hodis et al., 2012). RAC1, a RAS-related member of the Rho family of GTPases which regulate cytoskeleton rearrangements, contains the P29S substitution in around 4% of melanomas (Hodis et al., 2012). SKT19, a predicted kinase of known function, contains a D89N mutation in around 5% of melanomas.

Taken together, these recent *tour de force* studies reveal the complex array of mutations and genetic aberrations associated with melanoma genesis. Nevertheless it seems apparent that no other single mutation will have the same level of frequency as BRAF^V600E^, which is mutated in approximately 50% of human melanomas (Davies et al., 2002). Further analyses to discern driver from passenger mutations as well as their mechanisms of action are required to clarify the intervention targets and rational combination strategies likely to provide the most successful outcomes. What is abundantly clear, however, is that future therapies will require previous knowledge of the patient’s mutational status to guide the most appropriate intervention in a personalized fashion. So far only the targeted inhibitor of BRAF^V600E^ Vemurafenib has been approved for treatment of melanoma, however we foresee in the near future that an arsenal of therapies will be available based on the tumor genotype. Thus, it is envisaged that tumor specimens will in future, be subjected to targeted sequencing of all the potential mutation hot-spots for which there are therapeutic targets or which affect treatment outcome. However given the inter- and intra-tumor heterogeneity analysis of circulating melanoma cells may provide a comprehensive and sensitive tool for determining the overall mutation status of a patient’s tumors.

## Clinical Advances in Melanoma Targeted Therapies

### BRAF^V600E^ inhibitors

**Figure 1:**
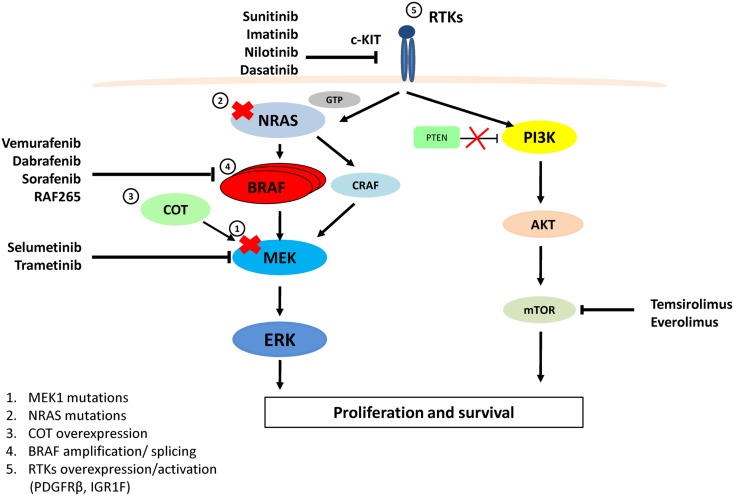
**MAPK and PI3K/AKT pathways, therapeutic targets for melanoma and resistance to Vemurafenib**. Vemurafenib and Dabrafenib are specific for BRAF^V600E^, while Sorafenib and RAF-265 are pan-RAF inhibitors. Imatinib, Nilotinib, Dasatinib, and Sunitinib target and inhibit c-KIT. Selumetinib and Trametinib inhibit MEK activity. Temsirolimus and Everolimus inhibit the mTOR protein. Resistance to Vemurafenib arises from MAPK pathway reactivation by (1) a MEK1^C121S^ mutation, (2) NRAS^Q61R/K^ mutations, (3) COT1 overexpression, (4) alternatively spliced variants of BRAF^V600E^ or amplification of the mutant BRAF allele, (5) Overexpression or activation of RTKs (PDGFRβ or IGF1R) bypasses mutant BRAF and activates ERK via CRAF-MEK or through independent ERK mechanisms by activating the PI3K/AKT pathway.

Developments in molecular targeted therapies (Figure 1; Table 1) have predominantly focused on targeting the BRAF, MEK, or c-KIT kinases located within the MAPK pathway. Two selective BRAF^V600E^ inhibitors Vemurafenib (commonly known as PLX4032, RG7204, or Zelboraf) and GSK2118436 (Dabrafenib) have demonstrated significant anti-tumor activity (Anforth et al., 2012; Falchook et al., 2012b; Long et al., 2012).

**Table 1:** **Anti-cancer inhibitors undergoing testing for treatment of cutaneous melanoma**.

Pathway	Treatment type	Target protein	Specific mutation	Trial	Effectiveness
MAPK	Vemurafenib (PLX4032)	BRAF	V600E/K	Phase I/II (Chapman et al., 2011) NCT01006980 (completed)	CR–PR = 1.8–98% RR = 48% PFS = 5.3 months OSR = 84% at 6 months
				Phase III – (updated BRIM-3) (Chapman et al., 2012)	RR = 48.4% Hazard ratio PFS = 0.26 (95% CI 0.2–0.33)
				NCT01006980 (ongoing study) Phase II (Sosman et al., 2012) NCT00949702 (completed)	OSR = 55% at 13.2 months CR–PR = 6–47% OR = 53% PFS = 6.8 months OS = 15.9 months
				Phase II NCT01586195 (recruiting participants)	N/A
				Phase II NCT01474551 (recruiting participants)	N/A
	
	Dabrafenib (GSK2118436)	BRAF	V600E/K	Phase I (Falchook et al., 2012b) NCT00880321 (completed)	CR–PR = 50–70% RR = 69% PFS = 5.6 months OSR = 47% > 6 months
				Phase II (Long et al., 2012) NCT01266967 (ongoing study)	PFS = 4 months OS > 7.8 months
				Phase II NCT01153763 (ongoing study)	N/A
				Phase III (Hauschild et al., 2012) NCT01227889 (ongoing study)	CR–PR = 3–47% OR = 50% PFS = 5.1 months Hazard ratio OS = 0.61 (95% CI 0.25–1.48)
	
	Sorafenib (BAY43-9006, Nexavar)	ARAF, BRAF, CRAF, VEGF2/3, KIT PDGFR	Not specified	Phase I (Pecuchet et al., 2012) (Completed)	OR = 21% at 10 months PFS = 3.6 months OSR = 33% at 11 months
				Phase I NCT01303341 (recruiting participants)	N/A
				Phase I NCT00565968 (recruiting participants)	N/A
				Phase I NCT01078961 (recruiting participants)	N/A
	
	RAF-265 (CHIR-265)	ARAF, BRAF, CRAF, VEGFR	Not specified	Phase I/II NCT00304525 (ongoing study)	N/A
				Phase Ib NCT01352273 (ongoing study)	N/A
	
	Selumetinib (AZD6244, PD0325901)	MEK	BRAF V600E	Phase II NCT00888134 (ongoing study)	N/A
				Phase II NCT00936221 (ongoing study)	N/A
				Phase II NCT01519427 (recruiting participants)	N/A
	
	Trametinib (GSK1120212, JTP-74057)	MEK	BRAF V600E/K	Phase II (Kim et al., 2011) NCT01037127 (ongoing study)	CR–PR = 4–30% SD = 47% RR = 33%
				Phase III (METRIC) (Robert et al., 2012) NCT01245062 (ongoing study)	OR = 24% PFS = 4.8 months Hazard ratio OS = 0.53 (95% CI 0.3–0.94)
				Phase I/II trial NCT01584648 (recruiting participants)	N/A
				Phase II NCT01619774 (recruiting participants)	N/A
				Phase III NCT01597908 (recruiting participants)	N/A

PI3K/AKT	Sunitinib (CGP57148, Gleevec, Glivec)	c-KIT	Not specified	Phase I/II NCT00859326 (recruiting status unknown)	N/A
	
	Imatinib (ST1571)	c-KIT	Not specified	Phase II NCT00424515 (ongoing study)	N/A
				Phase II NCT00470470 (recruiting participants)	N/A
	
	Nilotinib (AMN107)	c-KIT	Not specified	Phase II NCT01168050 (recruiting participants)	N/A
				Phase II NCT01099514 (recruiting participants)	N/A
	
	Dasatinib (BMS-354825, Bosulif, Sprycel)	c-KIT	KIT exon 11 and 13	Phase II NCT01092728 (recruiting participants)	N/A
	
	Temsirolimus (CCI-779)	mTOR	Not specified	Phase II (Margolin et al., 2005) California cancer consortium (completed)	N/A
				Phase II (Dronca et al., 2010) NCT00521001 (completed)	PR = 8% PFS = 2.4 months OS = 8.6 months
	
	Everolimus (RAD001)	mTOR	Not specified	Phase II NCT00976573 (recruiting participants)	N/A

Immuno-suppression blockage	Ipilimumab (MDX-010, BMS-734016)	CTLA-4	Not specified	Phase I (Hodi et al., 2010) NCT00094653 (completed)	CR–PR = 0–13% OR = 10.9% PFS ∼ 30% at 12 weeks OS = 23.5% 2 years
				Phase III (Robert et al., 2011) NCT00324155 (ongoing study)	CR–PR = 1.6–13.6% OR = 15.2% PFS ∼ 35% at 12 weeks OS = 28.5% 2 years
				Phase II (Di Giacomo et al., 2012) NCT01654692 (ongoing study)	CR–PR = 10–30% RR = 40% PFS > 5 months OS = 50% > 1 year
				Phase I/II NCT01400451 (recruiting participants)	N/A
	
	MDX-1106 (BMS-93558 or ONO-4538)	PD-1	Not specified	Phase Ib (Topalian et al., 2012) NCT00730639 (ongoing study)	CRR = 28% for 1 year PFS at 24 weeks = 41%
				Phase I NCT01621490 (recruiting participants)	N/A
				Phase I NCT01176474 (recruiting participants)	N/A
				Phase III NCT01721772 (recruiting participants)	N/A
	
	MK-3475	PD-1	Not specified	Phase I (Hamid, 2012) NCT01295827 (recruiting participants)	RR = 51% CR = 9%
	
	BMS-936559	PD-L1	Not specified	Phase I (Brahmer et al., 2012) NCT00729664 (recruiting participants)	OR = 17% PFS at 24 weeks = 42%

*PR, partial response; RR, response rate; CR, complete response; OR, overall response; PFS, progression-free survival; OS, overall survival; OSR, overall survival rate; SD, stable disease; N/A, data not available*.

*Vemurafenib* inhibits the mutant BRAF^V600E^ protein and also has inhibitory actions against other kinases, including CRAF, ARAF, and wild-type BRAF (Bollag et al., 2010). The phase III clinical trial (NCT01006980) compared the effect of oral Vemurafenib treatment (960 mg twice daily) to Dacarbazine (1000 mg/m^2^ intravenous every 3 weeks) in a total of 675 metastatic melanoma patients with the BRAF^V600E^ mutation. Response rates of more than 48% were observed in Vemurafenib treated patients compared to a 5% response rate in the Dacarbazine arm. The estimated median PFS (progression-free survival) for Vemurafenib was 5.3 months with an 84% overall survival at 6 months, compared to a median PFS of 1.6 months with a 64% overall survival at 6 months for Dacarbazine (Chapman et al., 2011). As a result of this study, Vemurafenib was approved by the US FDA in August 2011 as a new treatment standard for patients with unresectable or metastatic melanoma with a BRAF^V600E^ mutation (US Food and Drug Administration, 2011).

A separate phase II clinical trial of Vemurafenib treatment for patients with an activating BRAF^V600^ mutation (NCT00949702) included 132 previously treated melanoma patients. Patients were assessed for response rate, duration of the response, and overall survival after Vemurafenib treatment (Sosman et al., 2012). Patients received oral Vemurafenib at a dose of 960 mg twice daily. A complete response was reported in 6% (*n* = 8) of patients and a partial response was achieved in 47% (*n* = 62) of individuals with an overall response rate of 53%. Stable disease was noted in 29% (*n* = 38) of patients, while 14% (*n* = 18) of subjects demonstrated progressive disease. At the time of data analysis, patients demonstrated a median PFS of 6.8 months and an overall survival of 15.9 months (Sosman et al., 2012).

Common adverse events related to Vemurafenib treatment included fatigue, skin rash, joint pain, photosensitivity, nausea, and development of cutaneous squamous cell carcinomas (SCC) or keratoacanthoma (KA). From the 130 patients that presented adverse reactions to Vemurafenib, 34 patients developed a Grade III SSC or KA. More recently, Su et al. (2012a) reported the paradoxical activation of the MAPK pathway by Vemurafenib; Vemurafenib accelerates the growth of pre-existing cancerous lesions (SSC and KA) via upstream MAPK signaling, such as through HRAS^Q61L^ (Su et al., 2012a).

At the 2012 ASCO Annual Meeting, results were reported of the ongoing phase III (BRIM-3) randomized trial (NCT01006980) comparing Vemurafenib with Dacarbazine in previously untreated patients with BRAF^V600E^ melanomas (Chapman et al., 2012). In this trial, a total of 675 patients were randomly assigned to receive either 960 mg of oral Vemurafenib twice daily or Dacarbazine 1000 mg/m^2^ intravenously every 3 weeks. The median overall survival with Vemurafenib was 13.2 months compared to 9.6 months with Dacarbazine. The 12-month overall survival rates were 55% for Vemurafenib and 43% for the Dacarbazine patients. The hazard ratio for death was 0.62 in favor of the Vemurafenib patients. This study confirms the finding that a targeted therapy, Vemurafenib, improves overall survival rates for patients relative to treatment with a chemotherapeutic agent, Dacarbazine (Chapman et al., 2012).

*Dabrafenib* (GSK2118436) is a reversible, potent ATP-competitive inhibitor that blocks BRAF^V600E^ kinase fivefold more effectively that it does CRAF or wild-type BRAF. A phase I dose-escalation trial (NCT00880321) reported active inhibition of melanoma and brain metastases in response to Dabrafenib treatment (Falchook et al., 2012b). A total of 156 patients with metastatic melanoma were involved in the study; 3 of these patients were BRAF wild-type with the other 153 presenting with various BRAF^V600^ mutations. Overall, 47% of metastatic melanoma patients with a BRAF^V600E^ mutation maintained successful treatment for more than 6 months. A partial or complete response to Dabrafenib (dosage of 150 mg twice daily) was also noted in 18 BRAF^V600K^ mutation positive melanoma patients who were given varied doses of Dabrafenib (100–150 mg either once daily or twice daily). Of these, 39% (*n* = 7) demonstrated a partial response to treatment and 22% (*n* = 4) had a complete response to treatment. The median PFS for eight patients receiving Dabrafenib 150 mg twice daily was 5.6 months. For three patients with wild-type BRAF, PFS was 1.5 months. The PFS for patients presenting complex BRAF mutations (K601 and V600-K601insdelE) was 1.8 months. For BRAF^V600E^ patients who did not respond to treatment, PFS was 4.2 month. This study found Dabrafenib to be an effective inhibitor of mutant BRAF^V600E/K^ in metastatic melanoma patients with brain metastases and other solid tumors (Falchook et al., 2012b).

A follow-up phase II multicenter trial (NCT01266967) was conducted over six countries, with a total enrollment of 172 metastatic melanoma patients with confirmed BRAF^V600E^ (*n* = 139, 81%) or BRAF^V600K^ (*n* = 33, 19%) mutations and a brain metastasis. Patients were divided into two cohorts: cohort A consisted of patients who had not received previous treatment for brain metastases and cohort B, subjects had progressive brain metastases after previous treatment. All patients received 150 mg of oral Dabrafenib twice daily. In both BRAF^V600E^ and BRAF^V600K^ patients, overall survival was greater than 7.8 months. Interestingly, the overall response was lower amongst patients with a BRAF^V600K^ melanoma than it was in BRAF^V600E^ patients. For example, in cohort A intracranial responses were achieved in 39.2% (*n* = 29) of BRAF^V600E^ patients compared to the 6.7% (*n* = 1) response obtained in BRAF^V600K^ melanomas (Long et al., 2012).

An ongoing phase III randomized controlled trial (NCT01227889) reported recently showed an overall improved PFS for patients with BRAF^V600E^ mutant metastatic melanoma treated with Dabrafenib compared with Dacarbazine (Hauschild et al., 2012). A total of 187 patients received Dabrafenib (150 mg twice daily) and 63 patients were given intravenous Dacarbazine (1000 mg/m2 every 3 weeks). The median PFS for the Dabrafenib patients was 5.1 months compared to 2.7 months for the Dacarbazine patients. The complete response rate for the Dabrafenib patients was 3% (*n* = 6) compared with a 2% (*n* = 1) response rate for the Dacarbazine group. A 47% (*n* = 87) partial response rate was reported for the Dabrafenib subjects with a 5% (*n* = 3) partial response rate observed in the Dacarbazine group. As this clinical study is ongoing, the current overall survival hazard ratio reported is 0.61 (95% CI 0.25–1.48) in favor of Dabrafenib (Hauschild et al., 2012) but in contrast to the Vemurafenib phase III trial, in this trial all patients randomized to Dacarbazine were given the opportunity to cross over to Dabrafenib on progression masking any overall survival difference. Interestingly, Dabrafenib treatment showed less phototoxic reactions and proliferative epidermal lesions (SCC and KA) in only 6% of patients, compared to 11% under Vemurafenib treatment. On the other hand, inflammatory syndromes with fever, rare with Vemurafenib (6%), were recorded in 20% of Dabrafenib treated patients (Hauschild et al., 2012; Sosman et al., 2012).

Overall, treatment with Vemurafenib or Dabrafenib confers a survival advantage in metastatic melanoma patients and presents an encouraging treatment option. However, response to these two inhibitors is restricted to only a proportion of melanoma patients. Efforts to treat metastatic melanoma patients with broad spectrum multi-kinase inhibitors, as detailed below, would seem to be more broadly efficacious since they are independent of BRAF activating mutations, but in fact they are less so.

### Multi-kinase inhibitors

RAF multi-kinase inhibitor, *Sorafenib* (BAY 43-9006 or Nexavar), is an oral agent that inhibits many cellular targets including: VEGFR-2, platelet-derived growth factor receptor (PDGFR), c-KIT, FLT-3, CRAF, and BRAF. *In vitro* studies have demonstrated that Sorafenib induces cell cycle arrest and apoptosis in melanoma cell lines via MAPK activity inhibition (Gray-Schopfer et al., 2007). Sorafenib has been granted FDA approval for the treatment of advanced clear-cell renal carcinoma (Wilhelm et al., 2006), based on a randomized trial demonstrating prolonged PFS in patients (Escudier et al., 2007). However, it has demonstrated modest treatment outcomes in patients with advanced melanoma (Eisen et al., 2006; Flaherty, 2006). A recent phase II clinical trial (NCT00119249) confirmed that Sorafenib monotherapy had limited activity in patients with metastatic melanoma regardless of the BRAF^V600E^ mutational status of their tumor tissue (Ott et al., 2010). By contrast, a more recent study of 28 melanoma patients, showed that after 10 months follow-up there was a 21% overall response rate with a median PFS of 3.6 months and a 1-year survival rate of 33% (Pecuchet et al., 2012). Although Sorafenib has not shown increased PFS in melanoma patients there are ongoing clinical trials (NCT01303341, NCT00565968, and NCT01078961) currently recruiting participants which are investigating the effects of Sorafenib in combination with other treatments.

Broad spectrum kinase inhibitors *RAF-265* and *XL281*, known to target ARAF, BRAF, CRAF genes, and VEGFR receptors, have greater effectiveness and modestly improved selectivity for targeting BRAF compared with Sorafenib, in preclinical models and in patients with advanced solid tumors (Venetsanakos et al., 2006; Schwartz et al., 2009). A study using orthotropic implants of metastatic melanoma in mice, showed a 41% response rate, with more than 50% reduction in tumor growth after treatment with RAF-265 (Su et al., 2012b). Since the development of more potent BRAF inhibitors, clinical evaluation of RAF-265 inhibitor as a single-agent treatment for melanoma patients is not a strong focus. There is however, an ongoing phase I/II clinical trial (NCT00304525) evaluating the maximum tolerated dose of RAF-265 as an oral agent in patients with locally advanced or metastatic melanoma. Another ongoing phase Ib study (NCT01352273) is investigating the combination of the MEK inhibitor (MEK162) with RAF-265 in patients with advanced solid tumors harboring BRAF^V600E^ mutations and/or RAS mutations.

It is critical for the field of melanoma therapeutics, to enhance the longevity of the successful responses obtained with BRAF inhibitors. Therefore the focus now is on novel inhibitors designed to target other kinases within the MAPK pathway, for use individually or in combination strategies as additional treatment options.

### NRAS inhibition

Inhibition of NRAS has proven challenging as its GTPase activity has not allowed for successful design of specific small-molecule antagonists. RNA (siRNA)-mediated depletion of NRAS in two melanoma cell lines (224 and BL, which harbor a Q61R NRAS mutation) inhibits proliferation and renders cells more sensitive to chemotherapy (Eskandarpour et al., 2005). A single-agent, single-arm phase II trial conducted with metastatic melanoma patients investigated Farnesyltransferase inhibitors (FTI’s) which block farnesylation, the key activating post-translational modification of RAS (Sebti, 2005). The outcome of this trial using the FTI *Tipifarnib* (otherwise known as R115777), showed a low response in the first 14 patients which led to early closure of the trial (Gajewski et al., 2006). However, in this trial patients were not selected based on the presence or absence of NRAS mutations.

Due to the absence of successful specific RAS inhibitors for the treatment of melanoma, there are currently no registered clinical trials for the evaluation of NRAS inhibitors. Inhibition of RAS effector pathways would appear to be a more favorable option and investigations of these are underway. The next kinase in the pathway, MEK, has proven to be a more favorable target (Flaherty et al., 2012a).

### MEK inhibitors

*Selumetinib* (also known as AZD6244, ARRY-142886, or PD0325901) is a selective non-ATP-competitive inhibitor of the mitogen-activated protein/ERK kinase (MEK1/2) (Figure 1) (Davies et al., 2007). A successful early phase I trial with Selumetinib, opened the door for MEK inhibitors to be considered as efficacious for patients with metastatic cancer (Lorusso et al., 2005). In this phase I study, the BRAF status of most patients was unknown. However, two cases with known BRAF^V600E^ and one with an NRAS (not specified) mutation, displayed a positive response to treatment (Lorusso et al., 2005; Davies et al., 2007). A later phase II single-agent trial compared Selumetinib to Temozolomide. In this study of 100 genetically tested patients, 67 were BRAF and 24 were NRAS positive patients. Only six patients (five of them BRAF positive) receiving Selumetinib showed an 11% response rate. It is unclear why this trial showed such low response rates in patients and did not show a significant PFS rate relative to Temozolomide (Dummer et al., 2008). However a currently recruiting, phase II clinical trial (NCT01519427) will be investigating the efficacy of a combination of Selumetinib and the AKT inhibitor MK2206, for BRAF positive stage III and/or IV melanoma patients who had previously relapsed whilst on Vemurafenib or Dabrafenib treatment.

*MEK162* (also referred to as ARRY-162 or ARRY-438162) is a selective ATP-non-competitive inhibitor of MEK1/2 which inhibits the MEK protein as well as ERK phosphorylation in numerous cancer cell lines (Roberts and Der, 2007; Yeh et al., 2007). The phase I study of orally administered MEK162 in 28 patients with biliary tract cancer showed the drug was well tolerated and had clinical efficacy in patients. An 8% (*n* = 2 of 26 patients) overall response rate was observed in this study population. One patient was reported to have a complete response with a PFS of 8.1 months and another subject had a partial response to treatment with a PFS of 9.8 months. Overall 46% (*n* = 12) of patients had stable disease outcomes (Finn et al., 2012). Due to the overall positive response to treatment reported in this study, a phase II clinical trial (NCT01320085) investigating the safety and efficacy of MEK162 in patients with advanced or unresectable metastatic malignant melanoma, harboring BRAF^V600^ or NRAS mutations, is currently underway.

*Trametinib* (known as GSK1120212 or JTP-74057) is a selective oral MEK1/2 inhibitor which mediates blockage of the MAPK kinase MEK protein. Trametinib has been associated with improved PFS and overall survival in patients harboring BRAF^V600E/K^ mutations (Falchook et al., 2012a; Flaherty et al., 2012b). In a phase II trial (NCT01037127), patients harboring BRAF^V600E/K^ mutant melanoma were given 2 mg of oral Trametinib once daily. Of the patients who were previously treated with BRAF inhibitors (*n* = 40), 3% had complete response, 25% stable disease, and the median PFS was 1.8 months. By contrast, patients who previously received chemotherapy (*n* = 57), 4% had complete responses, 30% had partial responses, and 47% stable disease. This minimal activity observed in patients previously treated with BRAF inhibitors suggests that BRAF resistant mechanisms may also confer resistance to MEK inhibitor monotherapy (Kim et al., 2011).

Following this trial, an ongoing phase III randomized trial (NCT01245062) was initiated to investigate the efficacy of Trametinib compared to chemotherapy in patients with BRAF^V600E/K^ advanced or metastatic melanoma. Of the 322 enrolled patients, 214 received Trametinib while 108 received chemotherapy. At the time of analysis the confirmed overall response rate was 24% in the Trametinib patients and 7% in the chemotherapy group. A median PFS of 4.8 months for the Trametinib patients compared to 1.4 months for the chemotherapy patients was reported. The hazard ratio of overall survival was 0.53 (95% CI 0.30–0.94; *p* = 0.0181), favoring the Trametinib subjects. Frequent adverse events in the Trametinib patients included skin rash, diarrhea, edema, hypertension, and fatigue. This study found that, compared with chemotherapy, Trametinib provided a significant improvement in progression-free and overall survival for patients with metastatic melanoma (Robert et al., 2012).

### Combination targeted therapies

More recently, greater improvements have been noted in metastatic melanoma patients treated with *combination targeted therapies*, particularity so the combination of BRAF (Dabrafenib) and MEK (Trametinib) inhibitors (Flaherty et al., 2012a). When used as a single-agent Dabrafenib, like Vemurafenib, has shown patients developing resistance after approximately 5–7 months (Falchook et al., 2012b; Hauschild et al., 2012; Long et al., 2012). Therefore the rationale for adding a MEK inhibitor is that it may block the escape route for the BRAF inhibitor and allow continual response and remission in patients. The phase I clinical trial NCT01072175 tested the combination of oral Dabrafenib (150 mg) and Trametinib (2 mg) compared to Dabrafenib (150 mg) alone in 162 patients with metastatic melanoma containing the BRAF^V600E/K^ mutation. The Dabrafenib group (*n* = 54) had a median PFS of 5.8 months compared with a 9.4 month PFS in the combination group (*n* = 54) (Flaherty et al., 2012a). Currently the phase III trial (NCT01682083) is underway in metastatic BRAF^V600^ mutated melanoma patients comparing treatment combinations of Dabrafenib and Trametinib versus Dabrafenib alone. Other clinical trials currently recruiting patients with advanced or metastatic melanoma using the combination of Trametinib and Dabrafenib include NCT01619774, NCT01584648, NCT 01072175, and NCT01597908.

### KIT inhibitors

Activating mutations in c-KIT result in stimulation of the MAPK and PI3K-Akt pathways causing increased proliferation and survival advantages (Figure 1) (Webster et al., 2006). The c-KIT inhibitor *Sunitinib* is a potent inhibitor of mutant KIT with additional inhibitory effects on VEGF receptors (Chow and Eckhardt, 2007). A recent study conducted by Minor et al. showed that Sunitinib may provide a treatment option for melanoma patients with KIT mutations. Tumor tissues from 90 patients with stage III or IV acral, mucosal or cumulative sun-damaged melanoma were collected. The tumor tissues were sequenced for KIT, BRAF, NRAS, and GNAQ mutant genes and patients with amplification or overexpression of KIT were treated with Sunitinib. Of the melanoma patients treated with Sunitinib, 11% had mutations in KIT [other patients presented with mutations in BRAF (23%), NRAS (14%), or GNAQ (0%)]. Patients positive for KIT mutations (*n* = 4; exon 11; W557G, V559G, or L576P) showed varied responses to the treatment. One patient had complete remission for 15 months, while two patients demonstrated partial responses for 1 and 7 months respectively (Minor et al., 2012). A clinical trial (NCT00859326) is now in progress investigating the efficacy of a combination of Sunitinib and Temozolomide (an oral, cytotoxic chemotherapy agent) for the treatment of metastatic and unresectable malignant melanoma patients.

*Imatinib or Imatinib mesylate* (also known as ST1571, Gleevec, or Glivec) is a receptor protein kinase inhibitor targeting Abl, c-KIT, and the PDGFR (Fecher et al., 2007; Stuart and Sellers, 2009). In two phase II trials in patients with metastatic melanoma, Imatinib has shown no response and poor survival outcomes in 16 and 25 patients, respectively (Ugurel et al., 2005; Wyman et al., 2006). In contrast, a case report revealed that Imatinib may be an effective treatment, since in one patient with a c-KIT mutation in exon 11, a positive outcome to the treatment was observed (Hodi et al., 2008).

More recently the Imatinib inhibitor has been evaluated as a treatment option in melanoma patients presenting c-KIT mutations (Carvajal et al., 2011; Guo et al., 2011). The phase II trial, in 46 metastatic melanoma patients with c-KIT mutations or amplifications, demonstrated an overall response rate of 23.3%. All patients received a continuous dose of 400 mg of Imatinib, unless toxicities or disease progression occurred. Fifteen patients who experienced reoccurrence were given an increased dose of 800 mg per day. The median PFS for the 46 patients was 3.5 months, with a 6-month PFS rate of 36.6%, and an overall 1-year survival rate of 51%. The overall rate of disease control was 53.5%. This study found that Imatinib increased the overall PFS rate, response rate, and overall survival rate in patients presenting c-KIT mutations in exon 11 and 13. However, patients who had increased doses of Imatinib did not show improvements in disease control (Guo et al., 2011). Ongoing, is the phase II clinical trial (NCT00470470), investigating Imatinib in patients with unresectable stage III or IV melanoma harboring somatic alterations of c-KIT.

*Nilotinib* (also known as AMN107) is a second generation tyrosine kinase inhibitor known to inhibit KIT, PDGFR, and Bcr-Abl. It was approved by the FDA in 2010 for the treatment of chronic myeloid leukemia (CML) and has a similar target profile to Imatinib (Manley et al., 2010). A phase I clinical trial demonstrated that Nilotinib activity is safe and effective in CML resistant to treatment with Imatinib (Kantarjian et al., 2006) and a major clinical response was observed to Imatinib in KIT-mutated metastatic rectal melanoma (Hodi et al., 2008). A current clinical trial (NCT01168050) is examining Nilotinib as a first or second line treatment of primary melanoma, stage III unresectable, or stage IV melanomas with c-KIT mutations or amplifications (NILOMEL). Another clinical trial (NCT01099514) will also be investigating Nilotinib in metastatic melanoma with KIT aberrations.

*Dasatinib* (also known as Bosulif, Sprycel, or BMS-354825) is a tyrosine kinase inhibitor responsible for inhibiting src family kinases (c-src, yes, lck, and fyn), Bcr-Abl, c-KIT, PDGFβ receptor, and EPHA2 (Lombardo et al., 2004). Dasatinib was approved by the FDA for CML and gastrointestinal stromal tumors (GIST) (von Mehren, 2006; Pavlu and Marin, 2009). A recent single-arm phase II study of Dasatinib recruited 17 patients with advanced melanoma. The objective response rate was 5% with evidence of tumor regression after only four cycles of therapy (*n* = 5). The median PFS was 8 weeks. This study revealed that Dasatinib had limited activity in patients with advanced or unresectable melanoma and did not meet the pre-specified response rate (30%) or the 6-month PFS (Kluger et al., 2011). However a clinical trial (NCT01092728) is currently recruiting participants to investigate Dasatinib monotherapy in patients with acral lentiginous mucosal or chronic sun-damaged cutaneous melanoma.

### mTOR inhibitors

The therapeutic value of targeting the PI3K/AKT pathway in melanoma has not been as clearly elucidated as it has been for the MAPK pathway. However, it is clear that an active cross-talk between these two pathways supports the development of melanoma and leads to resistance to BRAF inhibitors. Due to the lack of PI3K and AKT inhibitors currently available for clinical trial evaluations in melanoma, attention has turned to mTOR for which several inhibitors are under development.

*Temsirolimus*, an mTOR inhibitor (also known as CCI-779), is an analog of Sirolimus (rapamycin) that has demonstrated immunosuppressive activity against melanoma in preclinical models and revealed benefits in patients with breast and renal carcinoma (Hidalgo and Rowinsky, 2000; Huang and Houghton, 2003; Lu et al., 2003). By contrast, an early study demonstrated that Temsirolimus activity resulted in poor clinical responses and limited disease PFS rates in metastatic melanoma patients (Margolin et al., 2005).

While this mTOR inhibitor study diminishes the therapeutic value of targeting the PI3K pathway in melanoma, preclinical evidence has shown, however, that co-targeting this pathway along with the MAPK pathway remains an important therapeutic option (Meier et al., 2007). For example, both PI3K and mTOR inhibitors have revealed synergistic responses when used in combination therapies with Sorafenib or MEK inhibitors (Molhoek et al., 2005; Meier et al., 2007; Lasithiotakis et al., 2008; Chappell et al., 2011). Interestingly the same response has not been generated with BRAF inhibitors (Meier et al., 2005; Molhoek et al., 2005). Current phase I/II clinical trials (NCT00281957, NCT01614301, and NCT01565837) investigating combination treatments which include Sorafenib, MEK inhibitors, chemotherapy agents, and stereotactic ablative radiation therapy along with Temsirolimus in patients with metastatic melanoma or advanced cancers are underway.

Another mTOR inhibitor *Everolimus* (also known as RAD001) is currently being investigated in patients with metastatic melanoma in the clinical trial NCT00976573, in which the chemotherapeutic agents (Carboplatin and Paclitaxel) and Bevacizumab are used with Everolimus. Another phase II study (NCT00521001) investigated the combination of Everolimus (10 mg daily, for 5 of 7 days) and Temozolomide (200 mg/m^2^ 1–5 days, every 28 days) in patients (*n* = 48) with stage IV metastatic melanoma. From the 48 patients, 8% (*n* = 4) achieved a partial response, the median PFS was 2.4 months and the overall survival was 8.6 months. The combination of Everolimus and Temozolomide did not offer a therapeutic advantage over Temozolomide alone (Dronca et al., 2010). However, a recent phase I study investigating the combination of Everolimus with Capecitabine in patients with advanced solid malignancies demonstrated a prolonged clinical benefit for 39% of patients (Deenen et al., 2012). Currently two clinical trials (NCT01252251 and NCT00976573) are investigating the therapeutic benefit of Everolimus treatment plus chemotherapy in patients with melanoma.

From the studies detailed above, it is clear that current and future clinical trials will focus on implementing several combination targeted therapies for melanoma patients in the hope of increasing survival rates and minimizing tumor regression. Since improved survival rates have been demonstrated in patients with advanced melanoma, particularly for Vemurafenib and Dabrafenib, trials are underway to develop novel inhibitors that target several genes within the MAPK pathways, as these can be used in combination targeted therapies with the hope of prolonging PFS. However this strategy is for patients with BRAF/NRAS/MEK mutations only. For patients with mutations in alternate pathways (PI3K and AKT) alternate therapies are required. The lack of efficacy to date, when alternate pathways are targeted may imply that combination treatments that also target the MAPK pathways, such as BRAF or MEK inhibitors together with an mTOR inhibitor, are required to prolong PFS and to prevent escape mutations. An alternate therapeutic option is immunotherapy, which is proving to be efficacious (Wilmott et al., 2012).

### Immunotherapy therapies

*Ipilimumab* (also known as Yervoy, MDX-010, or BMS-734016) a monoclonal antibody to the T-lymphocyte associated antigen 4 (CTLA-4) was approved by the US FDA in March 2011 and it is currently implemented as a treatment option for patients with stage III and IV metastatic melanoma. CTLA-4 is member of the immunoglobulin receptor family essential for the development of regulatory T-cells. Signaling through this molecule induces an inhibitory response that abrogates the cytotoxic response of the T-cells. Blocking this inhibitory signaling allows the tumor infiltrating lymphocytes to attack the tumors cells.

A phase III clinical trial (NCT00094653) reported by Hodi et al. (2010) demonstrated an improved survival rate in patients with unresectable stage III and IV melanoma. These patients received Ipilimumab either alone (*n* = 102) or in combination with the glycoprotein 100 peptide vaccine (gp100) (*n* = 403) (Hodi et al., 2010). In another phase III trial (NCT00324155) investigating Ipilimumab in combination with Dacarbazine for patients with previously untreated metastatic melanoma overall survival rates were 47.3% for 1-year, 28.5% for 2-years, and 20.8% for 3-years (Robert et al., 2011). This study demonstrated a slight improvement in the overall survival responses for patients who received Ipilimumab-plus Dacarbazine compared with patients who had received Ipilimumab-plus the gp100 vaccine. Di Giacomo et al. (2012) have reported on a more recent phase II clinical trial (NCT01654692) which assessed the combination of Ipilimumab and Fotemustine in patients with advanced, unresectable stage III or IV melanoma. A total of 46.5% (*n* = 40) of the study population maintained a stable disease within 12 months and a median PFS of 5 months (Di Giacomo et al., 2012). More than 50% (*n* = 10) of patients with brain metastases survived longer than 12 months, compared to approximately 20% survival reported for patients undergoing radiotherapy or surgery (Eigentler et al., 2011). Currently a phase I/II clinical trial (NCT01400451) combining BRAF targeted therapy (Vemurafenib) with immunotherapy (Ipilimumab) is underway in subjects with BRAF^V600E/K^ metastatic melanoma as a strategy to prolong PFS.

Other immunotherapeutic agents currently being tested are antibodies that interfere with the PD-1 (programed death-1) and PD-L1 (PD-1 ligand). PD-1 is a key immune co-inhibitory receptor expressed by activated T-cells which mediate immuno-suppression. The primary function of PD-1 is in peripheral tissue where T-cells encounter immunosuppressive ligands PD-L1 (also known as B7-H1 or CD274) and PD-L2 (also referred to as B7-DC or CD273) which are expressed by tumor and/or stromal cells (Dong et al., 1999; Menzies et al., 2012; Topalian et al., 2012). Anti-PD-1 antibodies interfere with the interactions between PD-1 and PD-L1 allowing the T-cells to attack the tumor cells (Iwai et al., 2002; Dong et al., 2003). The anti-PD-1 inhibitor monoclonal antibody *MDX-1106* (also referred to as BMS-936558 and ONO-4538) showed favorable preliminary evidence when administrated as a single-agent in a pilot study involving 39 patients with advanced solid tumors (Brahmer et al., 2010). In another study amongst participants with melanoma (*n* = 94), 28% (*n* = 26) had objective responses, lasting for 1 year or more (Topalian et al., 2012). Various trials are underway comparing the clinical benefit and overall survival after treatment with this anti-PD-1 antibody (NCT01621490, NCT01176474, and NCT01721772). *MK-3475* is another anti-PD-1 inhibitor which is being investigated in a phase I clinical trial (NCT01295827). Encouraging anti-tumor activity was reported at the recent Society for Melanoma Research Congress in November 2012 (Hamid, 2012). Objective anti-tumor responses were recorded in 51% (*n* = 43) of 85 patients analyzed to date. Of those 9% (*n* = 8) of patients demonstrated a complete response to MK-3475. Furthermore, a study conducted by Brahmer et al. (2012) has shown that the anti-PD-L1 antibody *BMS-936559* provides durable tumor responses in patients with advanced cancer including melanoma. These results validate the interaction between PD-1 and PD-L1 as an important target for therapeutic intervention in melanoma patients.

In general the anti-PD-1 and anti-PD-L1 treatments have achieved the highest rate of anti-tumor activity reported for an immunotherapeutic agent in the past 30 years (Ribas, 2012). Together with Ipilimumab, these immunotherapeutic agents have demonstrated an increased durability of the tumor response (Hodi et al., 2010; Brahmer et al., 2012; Topalian et al., 2012). Their low response rate compared to targeted therapies such as the BRAF^V600E^ inhibitors support their use in combination therapies. With two different modes of action, combination therapies that together target both cellular proliferation and immune response might provide enhanced inhibition of the spread of melanoma, and may overcome the development of drug resistance.

## Resistance to BRAF Inhibitors and Combination Therapies

Although there have been encouraging results with targeted BRAF inhibitors, such as Vemurafenib and Dabrafenib (Hauschild et al., 2012; Sosman et al., 2012), almost all patients on these therapies develop drug resistance after the initial response, leading to clinical relapse. The underlying reasons for the development of drug resistance can be found in the redundancy of molecular and cellular processes that mediate the development of melanoma (Figure 1). Significant efforts have been dedicated to the study of acquired resistance to BRAF inhibitors. Results from various groups indicate that resistance to BRAF inhibition can be attributed to a series of heterogeneous mechanisms that lead to the reactivation of the MAPK pathway. These mechanisms of reactivation include upregulation of NRAS through activating mutations (Q61K/R) (Nazarian et al., 2010), overexpression of COT/Tp12 by increased copy number of the *MAP3K8* locus (Johannessen et al., 2010), activation of MEK1 by mutation C121S (Wagle et al., 2011), alternative BRAF splicing (Poulikakos et al., 2011), or BRAF^V600E^ gene amplification (Shi et al., 2012b). Alternative, resistance is achieved by the activation of PI3K-AKT and RAS-CRAF-MEK pathways through receptor tyrosine kinase (RTK) signaling. Such activation includes, overexpression of platelet-derived growth factor β (PDGFβ) (Nazarian et al., 2010; Shi et al., 2012b) and activation of IGF1R (Villanueva et al., 2010). Interestingly, all these escape mechanisms are largely mutually exclusive and differ between patients and in some cases between tumors within a patient (Nazarian et al., 2010; Shi et al., 2012b).

### MEK mutation

Wagle et al. (2011) profiled tumors sensitive and resistant to BRAF inhibitors by massive parallel sequencing and identified the reactivation of the MAPK pathway by a newly identified mutation, MEK1^C121S^. MEK1^C121S^ also confers cross-resistance to the MEK inhibitor Selumetinib. However, this mutation has not been observed in any other studies of Vemurafenib-resistant tumors since then (Shi et al., 2012b). On the other hand, commonly found MEK exon 3 activating mutations such as MEK^P124S^ and MEK^I111S^ are shown to not confer Vemurafenib resistance (Shi et al., 2012a). Escape through a MEK activating mutation is therefore unusual and in contrast to most other mechanisms of acquired drug resistance, where the activation emerges downstream of the targeted kinase (Wagle et al., 2011).

### NRAS mutations

Nazarian et al. demonstrated that acquired resistance to Vemurafenib developed in melanoma cell lines and patient tumors by the acquisition of NRAS mutations. Interestingly, two biopsies from the same patient had two different activating NRAS mutations (NRAS^Q61R^ and NRAS^Q61K^) (Nazarian et al., 2010). More recently Shi et al. (2012b) reported that 5 of 15 patients with disease progression after responding to Vemurafenib, carried NRAS mutations. The NRAS mutated cells were sensitive to the MEK inhibitor, Selumetinib, in the presence or absence of Vemurafenib, suggesting that reactivation of the MAPK pathway might have occurred via CRAF bypassing the BRAF inhibition. This was later confirmed by re-sensitization of a cell line (NRAS^Q61K^/BRAF^V600E^) to Vemurafenib by knocking down CRAF expression (Shi et al., 2012b).

### COT overexpression

Through the screening of an “open reading frame” expression library encoding approximately 75% of the human kinases, Johannessen et al. (2010) identified that overexpression of COT/Tpl2 and CRAF reduced sensitivity to BRAF inhibitor PLX4720 (a preclinical version of Vemurafenib). Moreover, increased COT transcript levels were observed in two biopsies collected during Vemurafenib treatment and compared to lesion-matched pre-treatment biopsies. Furthermore, high levels of COT expression were related to an increased copy number of the *MAP3K8* locus in two cell lines resistant to PLX4720. Over-activation of MEK in the melanoma cell line A375 through COT signaling resulted in resistance to the MEK inhibitors Selumetinib and CI-1040. Nevertheless, the authors found that co-inhibition of both BRAF and MEK can overcome resistance to BRAF inhibitors caused by increased COT levels.

### Reactivation of BRAF

Contrary to intuition, no compensatory BRAF mutations have been found as a mechanism of resistance to BRAF inhibitors. However, reactivation of tumor progression after response to BRAF inhibitors can be achieved by tumor cells with an increased copy number of BRAF^V600E^. Indeed, Shi et al. (2012b) demonstrated that 20% of melanoma patients treated with BRAF inhibitors (Vemurafenib and Dabrafenib) showed an increase in genomic copy number of BRAF^V600E^ and BRAF^V600E^ amplification resulted in BRAF^V600E^ overexpression in tumors of melanoma patients whose cancer had progressed after initial response. Cell lines with BRAF^V600E^ gene amplification, thus resistant to BRAF inhibitors, remained sensitive to Selumetinib, with Vemurafenib and Selumetinib combination therapy producing a synergistic effect.

Poulikakos et al. identified BRAF^V600E^ splicing variants which lack a RAS-binding domain (RBD) in two cell lines. These cell lines displayed acquired resistance to Vemurafenib, that could not be explained by mechanisms previously described (Poulikakos et al., 2011). The observed truncated form of BRAF (p61BRAF) was the result of an in-frame deletion of exons 4–8. While the mechanism underlying this exon skipping phenomena remains to be identified, exons 4–8 encode domains essential for RAF activation, including the RBD and the cysteine-rich domain (CRD) (Wellbrock et al., 2004). The truncated BRAF lacking the RBD is able to dimerize independently of RAS signaling. Introduction of a mutation that abolishes p61BRAF dimerization restored sensitivity to Vemurafenib. Confirming this as a mechanism of resistance, BRAF variants lacking the RBDs were found in 6 of the 19 patients undergoing Vemurafenib treatment (Poulikakos et al., 2011), while Shi et al. (2012b) reported the same mechanism in another two patients. P61BRAF^V600E^ expressing cells remained sensitive to the MEK inhibitor Selumetinib. It is possible that these mechanisms of resistance may benefit from dose-escalation of the BRAF inhibitor, such as Dabrafenib, for which, the maximum tolerated dose has not yet been determined.

### RTK activation

In addition to the above mechanisms of acquired resistance to BRAF inhibitors, RTK overexpression or activation has been shown to bypass mutant BRAF and reactivate ERK through CRAF-MEK or via ERK independent mechanisms by activating the PI3K/AKT pathways. Upregulation of PDGFRβ and EGFR were demonstrated to mediate resistance to Vemurafenib developed in melanoma cell lines by Nazarian et al. (2010). In particular PDGFRβ displayed increased activation associated with tyrosine phosphorylation. Moreover the authors found that 4 of 11 post-relapse biopsies from melanoma patients treated with Vemurafenib showed increased expression of PDGFRβ in comparison to pre-treatment biopsies. The same increase was also observed in three relapse tumor biopsies from a patient treated with Dabrafenib (Shi et al., 2012b).

Platelet-derived growth factor receptor β knockdown by RNAi in resistant cell lines led to re-sensitization of the growth inhibition by Vemurafenib, but did not activate the apoptotic response (Nazarian et al., 2010). Thus, PDGFRβ overexpression might not be the only mechanism of resistance in these cells. Moreover, the PDGFRβ inhibitor Imatinib or the MEK inhibitor Selumetinib did not restore sensitivity to Vemurafenib (Shi et al., 2011). It is possible that resistance may involve the activation of more than one RTK.

BRAF inhibitor resistance also has been demonstrated to occur via phospho-activation of the RTK, IGF1R, with subsequent downstream activation of the PI3K/AKT pathways (Villanueva et al., 2010). Inhibition of IGF1R led to slower cell survival, but little improvement was observed when added in combination with the BRAF inhibitor. IGFR inhibition diminished pAKT activation, but did not suppress pMEK/pERK activation. Combination IGF1R inhibitor, PPP, with a MEK inhibitor, Trametinib, led to increased apoptosis and decreased cell viability (Villanueva et al., 2010).

Two recent reports showed RTK-mediated resistance to BRAF inhibition in colorectal carcinoma (Corcoran et al., 2012; Prahallad et al., 2012). Both studies showed activation of EGFR and downstream pathways (PI3K/AKT and MEK/ERK). All these studies underscore the role of RTK expression and activation in BRAF inhibitor acquired resistance. Given the redundancy and promiscuity of the RTKs signaling in melanoma cells, RTK reprograming might not effectively halt tumor growth. This leads to a proposition that co-targeting MEK, and the PIK/AKT/mTOR pathway would be a more effective strategy in response to this type of BRAF inhibitor induced resistance (Lo, 2012).

## Molecular Characterization of Circulating Tumor Cells for Personalized Treatment Monitoring

Targeted cancer therapies are effective in only a proportion of patients. For effective therapy accurate molecular analysis of a patient’s tumors is required, as incorrect administration can negatively impact on patient survival. Molecular tools are required that determine which patients are likely to benefit from the therapy and reveal, early during treatment, whether the therapy is effective. The quantification and molecular profiling of circulating tumor cells (CTCs) has been proposed as an aiding methodology for tumor genotyping and for early detection of therapy efficacy.

Several studies have investigated the value of detecting CTCs in melanoma patients by multimarker RT-PCR to predict response to therapeutic regimens with mixed outcomes. Reynolds et al. (2003) observed that therapy with a polyvalent melanoma vaccine was associated with clearance of melanoma cell markers (tyrosinase, gp100, MART-1, and MAGE-3) from the circulation and improved prognosis. Monitoring of CTCs by expression of five melanoma-associated biomarkers (*MART-1*, *GalNAc-T*, *PAX-3*, *MAGE-A3*, and *MITF*) in patients receiving biochemotherapy and maintenance biotherapy for stage IV melanoma suggests that CTCs detection may be useful for predicting therapeutic efficacy and disease outcome (Koyanagi et al., 2010; Reid et al., 2013). In a multivariate analysis, pre-treatment and serial CTC positivity (*MART-1*, *MAGE-A3*, and *PAX-3 RT-PCR*) was significantly associated with disease-free survival and overall survival (Hoshimoto et al., 2012) (NCT00052156). However, Fusi et al. (2012) reported that although CTCs positivity (*Mart-1 and tyrosinase*) was time dependant prognostic factor, it was not predictive of treatment outcome. Overall, CTC quantification using RT-PCR has been deemed prone to false positive results and the lack of validated and standardized methodologies has preclude its use as a biomarker in clinical trials (Nezos et al., 2009).

Several methodologies have been developed for cytometric detection of CTCs. At the fore front of these is the CellSearch system. Using this platform CTCs have been detected in cancer patients at both early and late stages, with the number of tumor cells in peripheral blood showing significant utility for prognosis in breast, colorectal, prostate, and non-small-cell lung cancers (Cristofanilli et al., 2004; Cohen et al., 2008; de Bono et al., 2008; Krebs et al., 2011). More recently, Khoja et al. demonstrated that CTCs were detectable in 40% of patients with advance cutaneous melanoma and the number of CTCs was prognostic for overall survival. They also showed preliminary evidence that changes in the number of CTCs during treatment may reflect outcome (Khoja et al., 2012). Currently additional trials are underway investigating the prognostic and predictive value of CTCs to identify responding patients treated with Ipilimumab (NCT01565837), Imatinib (NCT00470470), Everolimus (NCT00976573), and BRAF^V600E^ inhibitors (NCT01573494).

Circulating tumor cells not only constitute seeds for metastases and indicate the spread of the disease, but they also reflect the tumors within a patient, thus genetic changes in tumors could be readily detected in CTCs. Thus, CTCs could constitute an accessible sample with which to analyze the genetic profile of the tumors in a particular individual and possibly better represent the mutation status of all the tumors within a patient than a single biopsy. The detection of the BRAF^V600E^ mutation in CTCs isolated from melanoma patients has been previously reported (Kitago et al., 2009; Freeman et al., 2012). A recent report by Sakaizawa et al. (2012) successfully identified BRAF and KIT activating mutations at a single cell level in CTCs from patients with melanoma. Another study also showed the detection of BRAF^V600E^ in CTCs with a 91% (19/21) correspondence with the matched tumor tissue. Moreover, in one of those individuals CTCs were shown to bear the BRAF^V600E^ mutation while this was not present at the tissue level, again suggesting that the CTCs reflect the heterogeneity of the tumors (Fusi et al., 2011). This is consistent with previous observations of intra- and inter-tumor heterogeneity of BRAF mutation status in melanoma (Sensi et al., 2006; Yancovitz et al., 2012). Inter- and intra-tumor heterogeneity have been identified in several tumor types and it has been shown to affect responses to targeted therapies in GIST and lung cancer (Liegl et al., 2008; Taniguchi et al., 2008). Given the diverse clinical responses of melanoma patients to BRAF inhibitors, studies on the association between tumor heterogeneity and clinical outcome are needed. In this context, CTCs could constitute an accessible sample with which to analyze the genetic profile of the tumors in a particular individual and possibly better represent the mutation status of all the tumors within a patient than a single biopsy.

Molecular characterization of CTCs for personalized treatment monitoring has been demonstrated in other tumors besides melanoma. For example Maheswaran et al. (2008) described a successful molecular analysis of CTCs from patients with metastatic non-small-cell lung cancer. The drug resistance mutation T790M was detected in CTCs collected from patients with EGFR mutations that had received tyrosine kinase inhibitors Gefitinib (Iressa) or Erlotinib (Tarceva). The presence of the mutation correlated with reduced PFS from 16.5 to 7.7 months (*p* < 0.001). This result supports the idea of monitoring changes in tumor genotypes during the course of treatment, by genotyping CTCs. Similarly, the presence of KRAS mutations in EGFR-positive colorectal cancer partially explains why these tumors do not respond to anti-EGFR mAb Cetuximab (Erbitux). Molecular analysis of the primary tumor determines the suitability of this targeted therapy, however discordances in the KRAS mutational status between the primary and metastatic tumors have been reported in a small subset of patients with metastatic colorectal cancer (Artale et al., 2008; Italiano et al., 2010). This could explain the observed resistance in some patients despite having a wild-type KRAS primary tumor. Yang et al. (2010) detected the KRAS mutation in blood CTCs and suggested that the blood might be a better sample to assess the tumor genotype for treatment decisions.

Chromosomal amplification of androgen receptor (AR), rearrangement of ERG gene, PTEN deletion, and MYC amplification were detected in CTCs from patients with metastatic prostate cancer by FISH (Attard et al., 2009; Leversha et al., 2009). Moreover, Attard and colleagues demonstrated that CTCs, metastases and prostate tissue invariably had the same ERG gene status in therapy-naive prostate cancer patients. However, significant heterogeneity of AR copy number gain and PTEN loss were observed in CTCs, illustrating the heterogeneity of the tumors and the representation of this diversity in CTCs.

Altogether these observations support CTCs as a superior sample with which to examine the genetic profile of the sum of the patient’s tumors and may therefore be useful for monitoring the development of escape mutations during treatment. Nevertheless, prior studies that isolate and analyze CTCs are limited in that they concentrate on methodologies that utilize only one or two surface proteins, gene deletions, amplifications, or point mutations. More comprehensive studies are required that determine the extent to which CTCs represent the parental tumors. The rapid progress in next generation sequencing and onco-proteomics will enable in the near future, better characterization of CTCs. Hopefully this will uncover more informative biomarkers with which to select CTCs and thus provide more specific information about patients who will benefit from targeted treatments as well as improve evaluation of therapeutic responses.

In parallel, improvements in the methodologies used to isolate and quantify CTCs are needed. Different methodologies that bias toward different tumor cell subsets might not reflect the overall tumor(s) heterogeneity. Issues such as collective migration (microemboli), epithelial-mesenchymal transition (EMT), and metastatic potential of the CTCs still need to be addressed in the context of well designed clinical trials with highly sensitive molecular analyses to determine which procedures provide the best prediction of clinical treatment outcomes. It is likely that this will be different for different cancer types and therapeutic interventions. The use of CTCs as a companion to treatments is a valuable tool that should be evaluated as part of therapy clinical trials to facilitate a swift implementation into clinical practice.

## Conflict of Interest Statement

The authors declare that the research was conducted in the absence of any commercial or financial relationships that could be construed as a potential conflict of interest.
